# Physical Training and Healthy Diet Improved Bowel Symptoms, Quality of Life, and Fatigue in Children With Inflammatory Bowel Disease

**DOI:** 10.1097/MPG.0000000000003816

**Published:** 2023-05-03

**Authors:** Linda Elisabeth Scheffers, Iris K. Vos, E.M.W.J. Utens, G.C. Dieleman, S. Walet, J.C. Escher, L.E.M. van den Berg

**Affiliations:** From the * Department of Paediatric Gastroenterology, Erasmus MC—Sophia Children’s Hospital, Rotterdam, Netherlands; the † Respiratory Medicine and Allergology, Department of Paediatrics, University Medical Center, Erasmus MC—Sophia Children’s Hospital, Rotterdam, Netherlands; the ‡ Department of Paediatrics, Center for Lysosomal and Metabolic Diseases, Erasmus MC, Rotterdam, Netherlands; the § Department of Paediatric Cardiology, Erasmus MC—Sophia Children’s Hospital, Rotterdam, Netherlands; the ‖ Department of Child and Adolescent Psychiatry/Psychology, Erasmus MC—Sophia Children’s Hospital, Rotterdam, Netherlands; ¶ Research Institute of Child Development and Education, University of Amsterdam, Amsterdam, Netherlands; the # Department of Child and Adolescent Psychiatry, Amsterdam University Medical Center/Levvel, Amsterdam, Netherlands; the ** Division of Dietetics, Department of Internal Medicine, Erasmus MC, Rotterdam, Netherlands; the †† Department of Orthopedics and Sportsmedicine, Erasmus MC, Rotterdam, the Netherlands.

**Keywords:** Crohn disease, inflammatory bowel disease, pediatrics, physical exercise, ulcerative colitis

## Abstract

Physical activity programs have been suggested as adjunctive therapy in adult inflammatory bowel disease (IBD) patients. We assessed the effects of a 12-week lifestyle intervention in children with IBD.

**Methods:**

This study was a randomized semi-crossover controlled trial, investigating a 12-week lifestyle program (3 physical training sessions per week plus personalized healthy dietary advice) in children with IBD. Endpoints were physical fitness (maximal and submaximal exercise capacity, strength, and core stability), patient-reported outcomes (quality of life, fatigue, and fears for exercise), clinical disease activity (fecal calprotectin and disease activity scores), and nutritional status (energy balance and body composition). Change in maximal exercise capacity (peak VO_2_) was the primary endpoint; all others were secondary endpoints.

**Results:**

Fifteen patients (median age 15 [IQR: 12–16]) completed the program. At baseline, peak VO_2_ was reduced (median 73.3% [58.8–100.9] of predicted). After the 12-week program, compared to the control period, peak VO_2_ did not change significantly; exercise capacity measured by 6-minute walking test and core-stability did. While medical treatment remained unchanged, Pediatric Crohn's Disease Activity Index decreased significantly versus the control period (15 [3–25] vs 2.5 [0–5], *P* = 0.012), and fecal calprotectin also decreased significantly but not versus the control period. Quality of life (IMPACT-III) improved on 4 out of 6 domains and total score (+13 points) versus the control period. Parents-reported quality of life on the child health questionnaire and total fatigue score (PedsQoL Multidimensional Fatigue Scale) also improved significantly versus the control period.

**Conclusions:**

A 12-week lifestyle intervention improved bowel symptoms, quality of life, and fatigue in pediatric IBD patients.

## Graphical abstract

**Figure:**
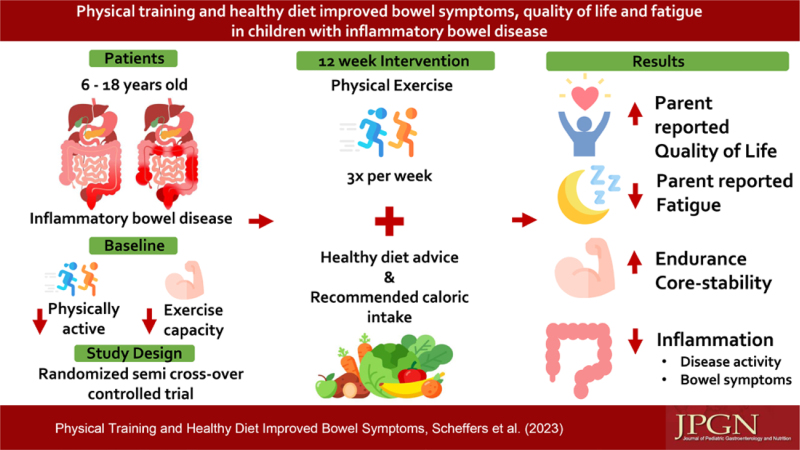

**What Is Known**

- Children with inflammatory bowel disease (IBD) have decreased levels of physical activity compared to healthy peers.
- Physical activity programs have been suggested as adjunctive therapy in adult IBD patients.

**What Is New**

- This study shows that a 12-week tailored physical training program including healthy dietary advice resulted in an increased submaximal exercise capacity and core stability, improved parent and self-reported quality of life, and less parent-reported fatigue.
- The intervention also seemed to have a positive effect on IBD as suggested by the lower clinical disease activity and fewer IBD symptoms.

Inflammatory bowel disease (IBD), including Crohn disease (CD) and ulcerative colitis (UC), are chronic inflammatory diseases of the gastrointestinal tract, characterized by periods of remission and relapse of symptoms (1). The most common symptoms include abdominal pain, severe diarrhea, and fatigue (2–4). Additionally, anxiety and depression are common in pediatric IBD patients (5,6). The current standard of care includes a combination of immunosuppressive, dietary treatment, and psychological support, and in some cases surgical intervention (1,7,8). Despite treatment options, many patients still suffer from disabling fatigue, which is associated with decreased quality of life (9). Recently, physical activity has been suggested as adjunctive therapy in adult IBD patients (10). Besides widely known favorable effects of physical activity on both physical and psychological health, it has also been reported to positively impact a variety of (auto-)inflammatory diseases (11). Pediatric IBD patients are less physically active and may have a reduced exercise capacity compared to healthy peers (12,13). Small studies in IBD patients have shown positive effects of exercise with reports of increased exercise capacity and quality of life, including positive effects on the clinical course of the disease (14). Currently, only 2 studies investigated the effects of physical activity in children with IBD (15,16). One study reported reduced inflammatory markers after 10 weeks of exercise gaming in pediatric IBD patients (15). Arruda et al showed reduced self-reported stress in children with IBD after 8 weeks of yoga (16). Lifestyle interventions (combined exercise and diet) in IBD patients have never been conducted before. This study aimed to assess the effects of a tailored lifestyle intervention on physical fitness (maximal and submaximal exercise capacity, strength, and core stability), the patient-reported outcomes (quality of life, fatigue, and fear), clinical disease activity, and nutritional status.

## METHODS

This was a prospective single-center randomized semi-crossover controlled trial, conducted between December 2019 and May 2021 at the Department of Pediatric Gastroenterology at Erasmus MC—Sophia Children’s Hospital in Rotterdam, The Netherlands. The trial was registered in the Dutch trial register: https://clinicaltrials.gov/, registration number: NL8181.

### Ethical Approval Statement

The study was performed in accordance with the Declaration of Helsinki and was approved by the Ethics Committee of Erasmus MC Medical Centre (NL.70912.078.19) and registered at https://trialsearch.who.int/Trial2.aspx?TrialID=NL8181 as Trial NL8181. Registration date: July 31, 2019, date of first enrollment: November 26, 2019. The protocol of the Exercise study was published before (17). All patients and parents signed informed consent.

### Patient Consent Statement

All patients and parents signed informed consent.

### Clinical Trial Registration

Ethics Committee of Erasmus MC Medical Centre (NL.70912.078.19), and registered at https://clinicaltrials.gov/ as Trial NL8181.

### Participants

Children, aged 6–8 years, with a diagnosis of IBD (CD, UC, or IBD-unclassified) confirmed by ileocolonoscopy and upper endoscopy with histology on multiple mucosal biopsies were eligible for enrollment. Exclusion criteria were: children with a physical inability to perform a cardiopulmonary exercise test (CPET), participation in organized exercise programs, and medical contra-indications for exercise.

### Study Design and Intervention

**FIGURE 1.**
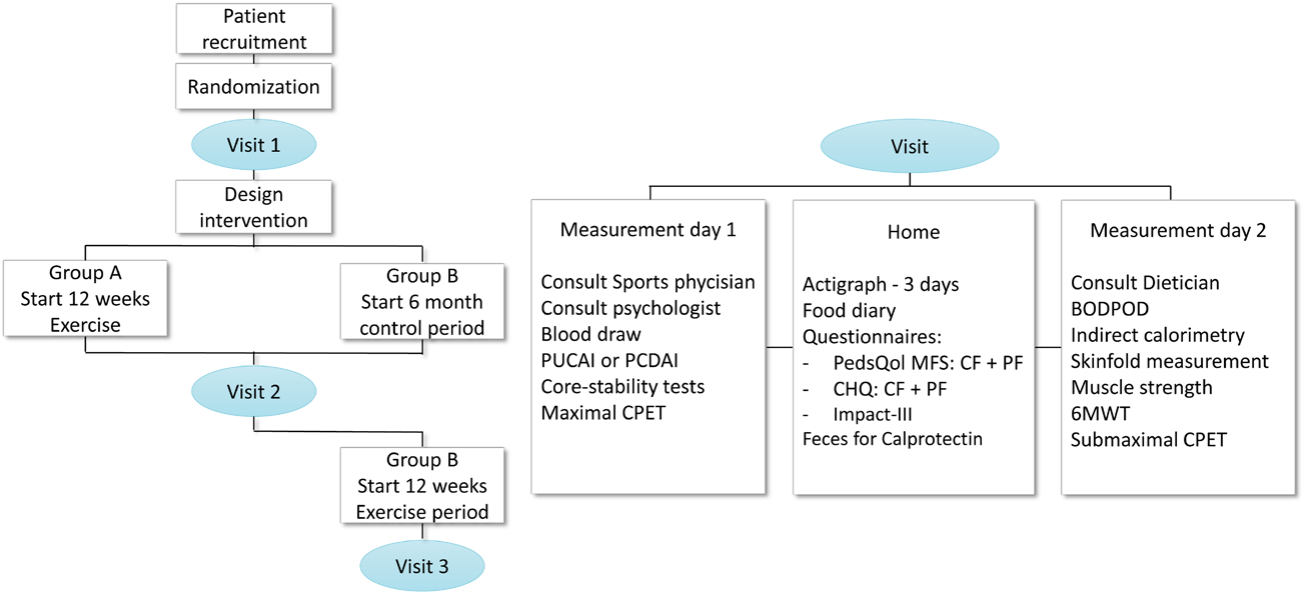
Study design and visits and measurement assessments. 6MWT = 6-minute walking test; BODPOD = body composition measurement system; CF = child form; CHQ = child health questionnaire; CPET = cardiopulmonary exercise test; MFS = Multidimensional Fatigue Scale; PCDAI = Pediatric Crohn disease Activity Index; PF = parents form; PUCAI = Pediatric Ulcerative Colitis Activity Index.

Figure 1 shows the study design, visits, and measurements. Children were randomized into group A (start exercise) or group B (start control period). Group A started the intervention immediately after the first assessment and did not have a control period. Group B started after a control period (this was planned to last for 6 weeks but due to the COVID-19 lockdown extended to 6 months). The tailored lifestyle intervention was designed as previously described (Supplement 1, Supplemental Digital Content, http://links.lww.com/MPG/D157) (17). The lifestyle intervention lasted 12 weeks, and consisted of 3 supervised training sessions (by a physiotherapist close to their home) per week, lasting 60 minutes each. The training program consisted of muscle endurance exercises (3 sets of 10–15 repetitions), and personalized aerobic training using heart rate (HR) zones 2, 3, and 4 based on HR at the anaerobic threshold (VT2) measured during the maximal CPET (full training program in Supplement 2, Supplemental Digital Content, http://links.lww.com/MPG/D157). Researcher L.E.S. visited the first training session to instruct the physical therapist and a training every 2 weeks to monitor uniform execution. Patients were telephoned weekly by L.E.S. to monitor side effects and assure compliance. In addition to the exercise program, all participants received a recommended caloric intake per day based on measured rest energy expenditure including a brochure regarding healthy diet in children (designed by the “Voedingscentrum,” the Dutch government-supported nutritional center) (18).

### Outcome Measurements

The primary study endpoint was the change in maximal exercise capacity measured by peak oxygen uptake (peak VO_2_); the golden standard for aerobic fitness.

Secondary study endpoints included:

(1) Physical fitness: submaximal exercise capacity, muscle strength, motor function, core stability, psychical activity levels.
(2) Patient-reported outcomes: quality of life, fatigue, and fear of exercise.
(3) Clinical disease activity: fecal calprotectin, blood draw, and validated disease activity scores.
(4) Nutritional status: caloric intake, energy balance, and body composition.

#### Physical Fitness

##### Exercise Capacity

Exercise capacity was assessed by maximal CPET and submaximal CPET using an electric brake bicycle ergometer, and a 6-minute walking test (6MWT). Details regarding CPET protocols can be found in our previously published exercise study protocol (1).

##### Muscle Strength and Core Stability

All muscle strength measurements were performed in a standardized manner by either L.J.G. or L.E.S. using hand-held dynamometry and compared to normal values of Beenakker et al (20). To assess core stability we measured time in balance for each of the following 4 core stability exercises: plank, back bridge, left side bridge, and right side bridge.

##### Physical Activity Levels

During the consultation with the sports physician, children and parents were asked about the amount of time spent on physical activity a week. Subsequently, physical activity levels were measured with a validated Actigraph GT3X+ accelerometer (firmware v3.2.1, ActiGraph Inc, Pensacola, FL) (17). The subjects were asked to wear the accelerometer on their right hip for 2 weekdays and 1 weekend day.

#### Patient-Reported Outcomes

##### Quality of Life, Fatigue, and Fear of Exercise

The validated child health questionnaire (CHQ) child form (CF) and parent form (PF) and the pediatric IBD-specific IMPACT-III questionnaire were used to assess health-related quality of life before and after the intervention (19). Higher scores indicate better quality of life. The PedsQoL Multidimensional Fatigue Scale (MFS) CF and PF were used to evaluate fatigue, with a higher score indicating less fatigue (20). During the semi-structured interview with the psychologist, children and parents were (separately) asked to score their fear of exercise on the fear thermometer, 0 (no fears at all) up to 8 (high fear of exercise).

### Clinical Disease Activity

To assess the effects of the intervention program on disease activity fecal calprotectin, laboratory measurements [c-reactive protein (CRP), erythrocyte sedimentation rate (ESR)], and clinical disease activity scores [Pediatric Crohn disease Activity Index (PCDAI) or Pediatric Ulcerative Colitis Activity Index (PUCAI)] were observed (21,22). Remission was defined as PCDAI < 10 (for CD) or PUCAI < 10 (for UC).

### Nutritional Status

#### Body Composition, Intake, and Energy Balance

Patient’s height and weight were measured, and body composition was assessed using a skinfold caliper (4 skinfolds method) and air displacement plethysmography on whole-body densitometry using the BOD POD (1). All patients filled in a detailed food diary for 3 consecutive days and underwent indirect calorimetry during the consult with the dietician to measure rest expenditure (REE). By multiplying REE by factors such as growth and activity, total energy expenditure (TEE) was calculated (12).

#### Sample Size Calculation and Statistical Analysis

In a previous Dutch study, untrained children with IBD had a mean peak VO_2_ of 36 mL/kg/min (12). Twelve IBD patients were needed to observe an increase in peak VO_2_ of at least 5% (clinically relevant), with a power of 80% and an alpha of 0.05 based on a standard deviation of 2.19 VO_2_/kg (23). Anticipated on a dropout rate of 30%–40%, 16 IBD patients were included. Data were collected in Castor EDC (Clinical Electronic Data Capture, 2019), and all analyses were performed using IBM SPSS Statistics 25.0 (IBM Corp, Armonk, NY). Patient characteristics were described using descriptive statistics. Baseline characteristics between groups were compared with the Mann-Whitney *U* and Chi-squared test for proportions. All data were analyzed as nonparametric due to the small sample size. Differences over the exercise and control period were analyzed using the Wilcoxon signed ranks test, and a generalized equations approach model was used to compare change between the periods and account for the correlation of the repeated measurements. The working correlation matrix was set as unstructured. The significance level was determined at *P* < 0.05.

## RESULTS

### Patient Characteristics

In total 50 subsequent patients visiting the clinic were asked to participate in the study by L.E.S. Given reasons not to participate were lack of time due to school obligations (n = 24), far distance from the hospital (n = 5), already performing sports 3 times a week (n = 3), and personal reasons (n = 2). A total of 16 patients were included in the study, age, gender, and distribution of disease were similar between included patients and patients who refused participation. In total, 15 patients completed the exercise intervention, 1 patient dropped out after 1 training session due to motivational problems. The median age of the patients was 15 years [12–16], 40% were female, and 67% were diagnosed with CD. Patient characteristics and maintenance treatments can be found in Table 1, which detailed information regarding medication use in Supplement 3, Supplemental Digital Content, http://links.lww.com/MPG/D157. Compliance for training was high, with a mean training session attendance of 93 ± 4.0%.

**TABLE 1.** Patient characteristics

	Study population	Group A (n = 8)	Group B (n = 7)	*P* values
	(n = 15)	Start exercise	Start control	Group A vs B
**Female, n (%**)	6 (40)	3 (37.5)	3 (43)	0.85
**Age, y**	15 [12 to 16]	15.5 [14.3 to 16.0]	12 [9 to 15]	0.07
**Age at diagnosis, y**	9.5 (4.0)	10.9 (4.2)	7.9 (3.3)	0.15
**Height for age, SDS**	−0.23 [−1.95 to 0.7]	−0.19 [−0.98 to 0.65]	−0.65 [−0.95 to 0.7]	1.00
**Weight for height, SDS**	0.95 [0.1 to 3.19]	1.52 [0.37 to 3.39]	0.21 [−0.38 to 0.95]	**0.04** *
**BMI, kg/m** ^2^	20.4 [17.5 to 31.5]	24.0 [18.5 to 32.4]	17.5 [16.3 to 22.3]	0.07
**Disease type (%**)				
** Ulcerative colitis**	5 (33.3)	1 (12.5)	4 (57)	0.07
** Crohn disease**	10 (66.7)	7 (87.5)	3 (43)	
**Extra intestinal manifestation (%**)				
** Arthralgia/arthritis**	3 (20)	2 (25)	1 (14.3)	
** Primary sclerosing cholangitis**	2 (13)	1 (12.5)	1 (14.3)	
** Active perianal fistulae**	1 (6.7)	1 (12.5)	0	
**COVID-19 positive during study (%**)	1 (6.7)	0	1 (14.3)	
**PCDAI + PUCAI**	15 [2.5 to 22.5]	10 [0.63 to 20.6]	15 [5 to 30]	0.08
**Maintenance treatment, n (%**)				
** Aminosalicylates**	4 (26.6)	3 (37.5)	1 (12.5)	
** Immunomodulators**	10 (66.7)	4 (50)	6 (85.7)	
** Biologicals**	10 (66.7)	5 (62.5)	5 (71.4)	
** Corticosteroids**	0	0	0^1^	
** No medication**	0	0	0	

Values are shown in mean ± SD or as median [IQR]. *P* values were measured using paired *t* testing (parametric data) or Wilcoxon rank sum test (nonparametric data). Difference between Group A and B were calculated using the Chi-square test or Fisher exact test. 1: One patient in group B received prednisolone during the exercise intervention, Supplement 2, Supplemental Digital Content, http://links.lww.com/MPG/D157 shows detailed medication descriptions including doses.

BMI = body weight index; PCDAI = Pediatric Crohn Disease Activity Index; PUCAI = Pediatric Ulcerative Colitis Activity Index; SDS = standard deviation score; vs = versus.

* *P* < 0.05.

### Physical Fitness

#### Exercise Capacity

Peak VO_2_ did not increase significantly. Watt_max_ and submaximal exercise capacity (time/wattage/VO_2_ at VT2) improved significantly (Table 2). Walking distance after training increased by an average of 40 meters (*P* < 0.001), and was the only one that increased significantly compared to the control period. During the submaximal CPET, average HR decreased but did not reach significance.

**TABLE 2.** Exercise capacity

	Exercise period (n = 15)			Control period (n = 7)				
	Before	After	*P* values	Before	After	*P* values	Effects size vs controls [95% CI]	*P* value Exercise vs control
Maximal CPET								
VO_2PEAK_, mL·min^–1^	2194 [1710–2901]	2286 [1800–2757]	0.532	1754 [1266–2641]	1679 [1060–2257]	0.499	114.1 [−227 to 455]	0.512
VO_2PEAK_, mL kg min^–1^	35.1 [28.6–47.8]	33.6 [25.7–49.6]	0.955	39.20 [29.82–50.21]	34.8 [30.1–43.1]	0.128	3.5 [−0.8 to 7.7]	0.113
Watt_MAX_, W	167 [125–203]	169 [137–221]	**0.033** *	124 [72–227]	133 [71–204]	0.933	13.3 [−10 to 37]	0.268
HR_PEAK_, beats· min^–1^	189 [180–196]	189 [183–192]	0.396	188 [179–191]	186 [180–189]	0.807	3.8 [−3.7 to 11.2]	0.321
Time_VT2_, min	8.3 [7.3–10]	9.4 [8.3–10.3]	**0.012** *	9.0 [7.0–9.3]	8.0 [6.3–10.0]	0.916	0.8 [−1.1 to 2.6]	0.423
Watt_VT2_, W	117 [82–134]	133 [81–162]	**0.009** *	95 [53–152]	121 [45–137]	1.000	17.6 [−12.1 to 47.3]	0.245
VO2_PEAK,VT2_, mL·min^–1^	1530 [1031–1701]	1719 [1022–1952]	**0.047** *	1209 [861–1863]	1515 [790–1701]	0.866	206.3 [−120 to 532]	0.215
HR_VT2_, beats min^–1^	163 [147–168]	163 [157–171]	0.267	159 [144–161]	163 [140–170]	0.933	5.1 [−11.7 to 22]	0.549
Submaximal CPET								
HR_average_, beats min^–1^	145 [137–151]	142 [136–154]	0.208	150 [146–161]	140 [137–156]	0.799	−0.1 [−13.3 to 13.1]	0.985
HR_PEAK_, beats min^–1^	155 [145–168]	153 [145–166]	0.233	160 [151–169]	149 [143–173]	0.866	−1 [−15 to 12.9]	0.884
6MWT								
Walked distance, m	480 [464–560]	536 [472–616]	**<0.001** *	511 [426–576]	536 [464–576]	0.113	25.5 [4 to 47]	**0.020** *

Values are shown as median [IQR]; effect size is shown with 95% confidence interval. *P* values were measured using paired *t* testing (parametric data) or Wilcoxon rank sum test (nonparametric data).

6MWT = 6-minute walking test; CPET = cardiopulmonary exercise test; HR = heart rate; kg = kilogram; Max = maximal; min = minutes; mL = millilitres; VO_2_ = oxygen uptake; VT2 = ventilatory anaerobic threshold; W = wattage.

* *P* < 0.05

#### Core Stability and Muscle Strength

Core stability improved significantly compared to the control period (Supplement 6, Supplemental Digital Content, http://links.lww.com/MPG/D157). Hip flexion increased significantly after training compared to the control period, the other muscle groups did not.

#### Physical Activity Levels

Five out of 16 children participated in sports activities before the COVID-19 lockdown. None of the children had to quit sports activities due to IBD-related symptoms earlier (<3 months). Median percentage of time spent in moderate-to-very vigorous activity measured with the Actigraph was 12.1% at baseline; this is below the recommended Dutch norm of 1 hour a day and did not change significantly after training (Supplement 7, Supplemental Digital Content, http://links.lww.com/MPG/D157).

### Patient-Reported Outcomes

#### Quality of Life, Fatigue, and Fear of Exercise

Quality of life, measured by the IBD-specific IMPACT-III questionnaire, improved on 4 domains out of 6 domains compared to the control period with an effect size of +13 points on the total score (Table 3). Children reported an increased quality of life in the general health and physical function domain of the CHQ, but not compared to the control period (Supplement 4, Supplemental Digital Content, http://links.lww.com/MPG/D157). Parents reported an increased quality of life in 8 domains, of which general health, physical functioning, and family cohesion improved significantly compared to the control period. Child-reported fatigue on the MFS did not change significantly, parent-reported fatigue did on the general fatigue and sleep/rest fatigue domains. Total MFS score improved significantly compared to the control period with an effect size of 14 points. Fear of exercise was low for both children and parents and sustained low (median score of 0).

**TABLE 3.** IMPACT-III and multi fatigue dimension scale

	Exercise period (n = 15 children, n = 24 parents)			Control period (n = 7 children, n = 12 parents)				
	Before	After	*P* values	Before	After	*P* values	Effects size vs controls	*P* value
							[95% CI]	Exercise vs control
IMPACT-III								
Total score	79 [71–81]	81 [75–89]	**0.006** *	79 [73–85]	70 [56–86]	0.108	13 [4 to 8]	**0.006** *
Bowel symptoms	75 [61–86]	82 [71–86]	**0.017** *	79 [64–82]	50 [46–93]	0.115	13 [1.3 to 25]	**0.029** *
Body image	67 [67–83]	75 [67–92]	0.258	67 [50–83]	67 [58–92]	0.276	17 [2 to 31]	0.679
Social functioning	83 [75–92]	88 [79–92]	0.173	81 [71–85]	73 [65–83]	0.498	9 [5 to 18]	**0.036** *
Systemic symptoms	67 [33–75]	75 [58–75]	0.147	58 [50–75]	67 [8–75]	0.350	−3 [−16 to 10]	0.433
Emotional functioning	82 [71–86]	86 [75–93]	0.100	82 [79–93]	79 [57–93]	0.138	13 [−19 to 44]	**0.029** *
Treatment	75 [67–92]	83 [83–100]	0.082	92 [67–100]	67 [42–83]	**0.026** *	26 [17 to 36]	**<0.001** *
MFS child report								
General fatigue	70.8 [50–79.2]	70.8 [66.7–79.2]	0.084	62.5 [50–83.3]	75 [33.3–95.8]	0.497	1 [−23 to 25]	0.945
Sleep/rest fatigue	58.3 [50–70]	66.7 [50–75]	0.346	70.8 [50–87.5]	62.5 [54.2–75]	0.345	5 [−11 to 22]	0.533
Cognitive fatigue	75 [55–91.7]	75 [66.7–87.5]	0.475	70.8 [66.7–91.7]	91.7 [55–91.7]	0.463	−5 [−20 to 10]	0.542
Total score	70.8 [50–79.2]	70.8 [66.7–79.2]	0.367	68.1 [56.9–87.5]	72.2 [55.6–87.5]	0.917	0 [−15 to 15]	0.990
MFS parent report								
General fatigue	56 [41.7–69.8]	70.8 [55.2–78.1]	**0.004** *	60.4 [54.2–72]	54.2 [31.3–74]	0.783	18 [−1.3 to 37]	0.069
Sleep/rest fatigue	54.2 [39.6 –72.9]	66.7 [50–82.3]	**0.044** *	69 [56.9–82.3]	56.3 [39.6–82.3]	0.126	13 [−2.4 to 28.4]	0.098
Cognitive fatigue	75 [53.1–94.8]	75 [54.2–95.8]	0.433	79.2 [75–100]	81.3 [56.3–100]	0.390	6.7 [−1.1 –14.4]	0.092
Total score	62.5 [49.7–74]	71.5 [58.7–82.3]	**0.015** *	68.2 [61.8–82.6]	64.6 [52.8–83.7]	0.533	14 [0.1 to 30]	**0.048** *

Values are shown in mean ± SD or as median [IQR]. *P* values were measured using paired *t* testing (parametric data) or Wilcoxon rank sum test (nonparametric data).

MFS = Multidimensional Fatigue Scale.

* = *P* < 0.05.

### Clinical Disease Activity

No disease exacerbations were observed during the training period. Supplement 5, Supplemental Digital Content, http://links.lww.com/MPG/D157 shows clinical disease activity outcomes. Fecal calprotectin decreased significantly after 12 weeks of training (400 µg/g [57.1–1662.7] vs 128 µg/g [23.8–642.3], *P* = 0.016), but not compared to the control period. PCDAI and PUCAI scores also decreased significantly, which remained significant compared to the control period for PCDAI (effect size of −19 points compared to the control period). Both ESR and CRP decreased but did not reach significance. The number of patients in clinical remission increased from 5 to 12 (*P* < 0.001), which was also significant compared to the control period. Medication use (Supplement 3, Supplemental Digital Content, http://links.lww.com/MPG/D157) during the study remained unchanged, except for 1 patient with ongoing active colitis (despite escalation of Vedolizumab infusion), who received a 4-week course of oral prednisolone as bridging treatment during the exercise period.

### Nutritional Status

#### Body Composition and Energy Balance

Median measured REE was increased (+14%) compared to healthy peers (Supplement 8, Supplemental Digital Content, http://links.lww.com/MPG/D157). Difference between caloric intake and recommended intake (TEE) based on measured REE was large (median 839 calories), and most children (n = 12) had to eat more calories. The absolute difference between caloric intake and recommended intake tended to become smaller; compared to the control period, body fat measured by skinfold increased during the exercise period (effect size +2.7%, *P* < 0.001).

## DISCUSSION

This study is the first to investigate the effects of a lifestyle intervention on a broad set of outcomes in children with IBD. The lifestyle intervention resulted in improved physical fitness, quality of life, and parent-reported fatigue and also seemed to have a positive effect on the IBD as suggested by the lower clinical disease activity accompanied by reports of fewer bowel symptoms.

### Physical Fitness

#### Exercise Capacity

At baseline, peak VO_2_ was decreased compared to healthy peers; this was in accordance with previously published studies, claiming that pediatric IBD patients suffer from reduced exercise capacity (12). After the intervention, exercise capacity measured by peak VO_2_ did not improve significantly, PeakVO_2_ at VT2 did. Peak VO_2_ at VT2 is therefore more representative of physical functioning during daily life activities. Compared to the control period, VT2 did not improve, as the control period was prolonged due to the COVID-19 lockdown; this is most likely caused by larger intra-patient differences and lack of power. To the best of our knowledge, only one other recently published study investigated the effects of exercise on exercise capacity measured by peak VO_2_ in adult IBD patients, which also did not improve (24). The improved walking distance during the 6MWT did remain significant compared to the control period.

#### Physical Activity Levels

The intervention did not increase physical activity levels measured with the Actigraph. This might be related to measurement timing since baseline measurements were performed before the COVID-19 pandemic and post-intervention measurements. In addition, children might have taken some time off from exercise during the week after 12 weeks of training.

### Patient-Reported Outcomes

#### Quality of Life, Fatigue, and Fear of Exercise

Children and adolescents with IBD experience decreased quality of life, increased anxiety and depression, and more fatigue compared to healthy peers (5,25). Both children and parents reported improvements in quality of life on the CHQ domain physical function. Only 1 prospective study investigated the effects of physical activity (yoga) on the quality of life in children with IBD; this study was underpowered to detect any change. The IMPACT-III questionnaire showed a significant and clinically relevant increase in 4 domains compared to the control period, including a large improvement in bowel-related symptoms (effect size +13 points, *P* = 0.029). While self-reported fatigue did not change significantly, parents reported a clinically important improvement in total fatigue score compared to the control period (+14 points, *P* = 0.048). The discrepancy between the parent’s and child reports can be partly explained by a lack of power in the child forms (parent reports n = 26, child reports n = 15).

### Clinical Disease Activity

After the lifestyle program, PCDAI scores and thereby number of patients in remission decreased significantly. This was mainly declared by a decrease in self-reported bowel symptoms on the PCDAI/PUCAI. Fecal calprotectin decreased, but not compared to the control period, mainly due to relatively large intra-patient fluctuations in the control period. As none of the participants experienced any side effects of training or exacerbation of disease, we think the intervention was safe; our sample size is too small to draw definite conclusions and longer-term effects remain unknown. Whether the decrease in clinical disease activity can be attenuated by our intervention is hard to verify. The only other long-term exercise intervention (8 weeks of exercise gaming) in pediatric IBD patients, also showed a decrease in inflammatory markers (15). The mechanism behind the anti-inflammatory effects of exercise has not been clarified. Multiple theories have been suggested in previously published studies such as a reduced release of adipokines due to less visceral fat, increased secretion of anti-inflammatory myokines such as interleukin (IL)-6, and reduced transient stool time (10,12,15,26).

### Nutritional Status

Although most children did not consume sufficient calories according to their food diaries, growth was comparable to healthy peers. Body fat measured over the control period (by both skinfold and BODPOD; body composition system, COSMED, Ltd, Concord, CA) decreased, leading to a significantly higher body fat percentage measured by skinfold over the exercise period compared to the control period. It has to be noted that one patient experienced an exacerbation of disease during the control period and lost 20 kilograms; she gained weight again during the exercise period. Whether children also gained muscle mass, is uncertain; REE as percentage of predicted tended to increase, indicating a higher muscle mass, but did not reach significance. Both the BODPOD and skinfold caliper only measure fat mass and fat-free mass; further studies may need to use dual X-ray absorptiometry to investigate training effects on muscle mass.

### Strengths and Limitations

Our study has several strengths. This study is the first to prospectively investigate the effects of a lifestyle intervention in pediatric IBD patients on a broad set of outcomes, which are never investigated before in this population. The program was well received, as reflected by the high training adherence, making this program feasible in a clinical setting. Whether patients were also adherent to the tailored diet advice, was hard to verify. A weakness of our study is the small study population, especially the control group (n = 7). Researchers in the study could not be blinded by following strict protocols and consulting a second reviewer when filling in the PCDAI/PUCAI questionnaires; this bias was minimized. Due to the invasive nature of the procedure, we did not measure mucosal inflammation by endoscopy before and after the intervention. A major limitation of our study is the prolonged control period due to the COVID-19 lockdown. Although we still executed the generalized estimation approach model to measure change during the control period versus the exercise period, comparing these periods is less accurate, as a 6-month control period leads to more intra-patient fluctuations, especially in a disease that is characterized by periods of remission and relapse.

## CONCLUSIONS

The 12-week lifestyle intervention resulted in improved physical fitness, quality of life, and parent-reported fatigue. In addition, a combination of lower clinical disease activity scores accompanied by fewer IBD symptoms suggests positive effects on intestinal inflammation. Children and adolescents with IBD should be motivated and supported to acquire and maintain a healthy lifestyle.

## Acknowledgments

We would like to thank all patients for participating in our trial and all physiotherapists for training the patients. Abir Bougrine & The Rotterdam Exercise Team: W.A. Helbing, M.W. Pijnenburg, A.T. van der Ploeg, J. Noske, A. van den Broek, and J. Olieman.

## Supplementary Material


