# Supplementary material for: Physical Training and Healthy Diet Improved Bowel Symptoms, Quality of Life, and Fatigue in Children With Inflammatory Bowel Disease
Source: J Pediatr Gastroenterol Nutr. 2023 May 3;77(2):214–21. doi: 10.1097/MPG.0000000000003816 (PMC10348627; doi:10.1097/MPG.0000000000003816)
Supplement: Supplementary file 1 [file mpg-77-214-s001.pdf]

Supplement 1– Flowchart design Tailored exercise intervention

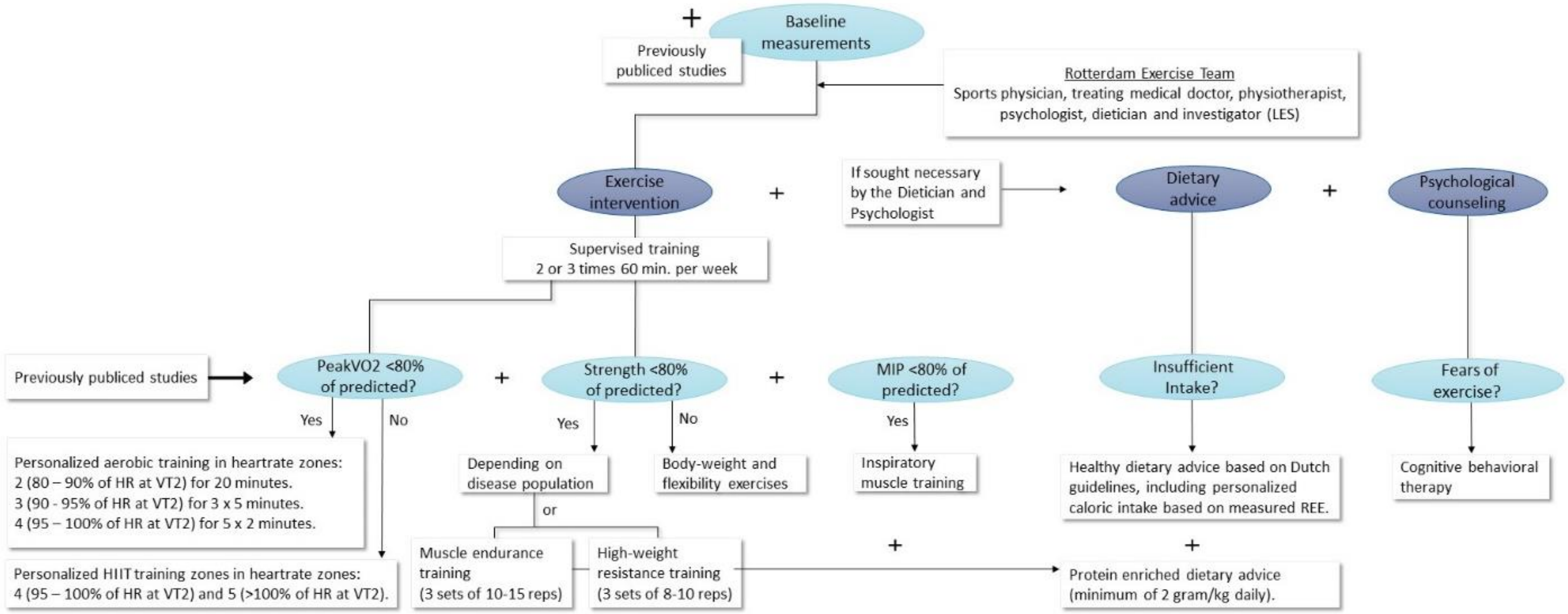

Supplement 1 shows how we designed the tailored training intervention. As patients showed a PeakVO2 of <80%, normal strenght and insufficient intake out program consisted of personalized aerobic training, body weight and flexibility exercises and a healthy diet advice including recommended caloric intake.

Supplement 2– Exercise intervention

Aerobic training

Performed on treadmill, ergometer, hometrainer or crosstrainer		
Training 1: Zone 3<br><br>90%-95% van HF VT2	Training 2: Zone 2<br><br>80%-90% van HF VT2	Training 3: Zone 5<br><br>95%-100% van HF VT2
3 x 5 minutes with 90 seconds break inbetween.	20 minutes without a break.	5x2 minutes with 30 seconds break inbetween.

*When a training was performed on a treadmill, ergometer, hometrainer, the heartratezones were increased with 10 beats. As the VT2 was determined during cycling, HRmax is known to remain lower.

Muscle endurance training

**Each exercise was performed in sets of 3 containing 10-15 repetitions each.<br>Once a child could perform 15 repetition, firstly the number of repetitions or seconds in balance was increased.<br>If a child could reach 30 repetitions (or 1 minute in balance) the exercise was made harder by increasing the weight or making the exercise more difficult (for example by making the exercise instable, as explained below).**		
**Level 1**	**Level 2**	**Level 3**
Side bridge Left,3 x 30 seconds	Side bridge Left, 3x 60 seconds	Side bridge Left, 3x 60 seconds<br><br>+ outstretched arm or leg, or bose ball.
Side bridge Right,3 x 30 seconds	Side bridge Right, 3x 60 seconds	Side bridge Right, 3x 60 seconds<br><br>+ outstretched arm or leg, or bose ball.
Back bridge, 3 x 30 seconds	Back bridge, 3 x 60 seconds	Back bridge, 3 x 60 seconds<br>+ outstretched arm or leg<br>+ performed on bose ball<br>+ weight on belly
Plank, 3x30 seconds	Plank, 3 x 60 seconds	Plank, 3 x 60 seconds<br>+ outstretched arms<br>+ weight on back<br>+ performed on bose ball
Superman exercise, 3x15 reps	Superman exercise, 3x30 reps	Superman exercise, 3x30 reps including weight in hands
Mountainclimber, 3x15 reps	Mountainclimber, 3x30 reps	Mountainclimber, 3x30 reps<br>+ performed on bose ball
Bicycle exercise, 3x15 reps	Bicycle exercise , 3x30 reps	Bicycle exercise -> sit ups<br>+ performed in high speed<br>+ performed including weight in hands
Wall-push up, 3x15 reps	Knee-push up, 3x15 reps	Push-up, 3x15 reps<br>+ performed on bose ball<br>+ weight on back
Squat, 3x15 reps	Squat, 3x30 reps	Squat, 3x30 reps<br>+ weight in hands

Reps (repetitions), HR (heartrate), VT2 (ventilator anaerobic threshold).

Supplement 3 – Maintenance treatment and Change in Medication usage during the Exercise study

Randomisation group	Maintenance treatment at start exercise	Changes in medication usage during and 3 months prior to the Exercise study
**Group A**		
Patient 1	6-Mercaptopurine 50 mg, qd<br>Ursodeoxycholic acid 600 mg, bid<br>Desloratadine 5mg, qd	No changes in medication
Patient 2	Infliximab i.v. 650 mg, q6weeks	De-escalation: Infliximab 650 mg, q8 weeks
Patient 3	Azathioprine 50 mg, qd<br>Mesalazine 200 mg, qd<br>Ursodeoxycholic acid 2x300 mg, qd<br>Infliximab i.v. 1000 mg, q4weeks	No changes in medication
Patient 4	Infliximab i.v. 500 mg, q6 weeks<br>Desloratadine 5 mg, qd	No changes in medication
Patient 5	Adalimumab s.c. 40 mg, q2weeks<br>Leflunomide 20 mg, qd	No changes in medication
Patient 6	Azathioprine 150 mg, qd	No changes in medication
Patient 7	Azathioprine 125 mg, qd<br>Adalimumab 40 mg, q1week	No changes in medication
Patient 8	Thiosix 15 mg, qd	No changes in medication
**Group B**		
Patient 9	Azathioprine 75 mg, q2weeks	No changes in medication
Patient 10	Cotrimoxazol 3 days, q1week<br>Vedolizumab i.v. 300 mg, q6weeks<br>Thiosix tablet 20mg, qd	During the control period the patient switched from infliximab -> vedolizumab 300 mg/ 8 weeks, this was 4 months prior to start Exercise.
Patient 11	Mesalazine 1 gram, bid<br>Ursodeoxycholic acid 500mg, tri	No changes in medication
Patient 12	Azathioprine 75mg qd<br>Infliximab 300 mg, q4weeks<br>Mesalazine 2x100 mg qd	Escalation of infliximab during the control period from q8 weeks to q4 weeks, 3 months prior to start Exercise.
Patient 13	Infliximab i.v. 510 mg, q6weeks	No changes in medication
Patient 14	Vedolizumab i.v. 200 mg, q6weeks<br>Mesalazine 200 mg, qd	Escalation of vedolizumab during the control period from q6 weeks to q4 weeks; During the Exercise period 30 mg prednisolone/day was given for 4 weeks due to pre-existing (before start exercise study) and persistent bloody diarrhea.
Patient 15	Adalimumab 40 mg, q1week<br>Ustekinumab 90 mg, q8weeks<br>Metoject 20 mg, q1week	During the control period, 5 months prior to start Exercise, this patient started on Ustekinumab.

Supplement table 4: Quality of Life measured by Child Health Questionnaire

	Exercise Period (n=15 children, n=24 parents)			Control Period (n=15 children, n=11 parents)				
	Before	After	P-values	Before	After	P-values	Effects size vs controls [95% CI]	P-value Exercise vs control
**CHQ child form**								
General Health	60 [30 - 60]	85 [60 – 85]	**0.036***	60 [60 - 85]	60 [30 -73]	0.734	15 [-9 – 39]	0.213
Physical Functioning	89 [72 – 100]	100 [89 – 100]	**0.010***	89 [78 – 94]	94 [389 – 100]	0.866	16 [-7 – 39]	0.117
Role/Social: Emotional	100 [100 – 100]	100 [67 – 100]	0.104	83 [83 – 100]	100 [66 – 100]	0.564	-13 [-27 – 1]	0.069
Role/Social: Physical	83 [66 – 100]	100 [67 – 100]	0.124	100 [66 – 100]	100 [58 – 100]	0.892	17 [-19 – 54]	0.348
Bodily Pain	60 [50 - 80]	80 [70 – 80]	0.081	60 [30 – 80]	80 [20 – 100]	0.893	12 [-19 – 44]	0.440
Behavior	79 [71 – 92]	83 [67 – 92]	0.304	88 [79 – 92]	75 [71 – 100]	0.092	7 [-3 – 17]	0.161
Global Behavior	85 [60 – 85]	85 [60 – 85]	0.605	85 [60 – 85]	85 [60 – 85]	1.000	-2 [-16 – 11]	0.729
Mental Health	72 [64 – 88]	75 [67 - 86]	0.807	78 [69 – 81]	72 [64 – 94]	1.000	1 [-12 – 13]	0.513
Self Esteem	79 [64 – 83]	75 [71 – 93]	0.752	71 [64 - 96]	82 [75 – 83]	0.735	5 [-9 – 19]	0.926
General Health perceptions	63 [44 – 81]	63 [44 – 75]	1.00	31 [25 -56]	63 [44 – 81]	0.104	-27 [-52 - -2]	0.077
Change in Health	5 [3 – 5]	5 [4 – 5]	0.457	2 [1 – 5]	4 [2 – 5]	0.673	-1 [-3 – 1]	0.336
Family Activities	100 [75 – 100]	100 [92 – 100]	0.262	100 [75 – 100]	75 [50 – 100]	0.141	13 [-17 – 44]	0.339
Family Cohesion	85 [60 – 85]	85 [60 – 100]	0.779	85 [60 – 100]	85 [60 – 100]	0.705	-6 [-25 – 12]	0.505
**CHQ parent form**								
General Health	60 [30 – 60]	60 [60 – 78.8]	**0.005***	60 [30 – 60]	60 [30 – 60]	0.739	21 [1 – 41]	**0.036***
Physical Functioning	78 [39 – 89]	100 [88.7 – 100]	**0.001***	89 [77 – 94]	89 [44 – 100]	0.673	36 [10 – 62]	**0.007***
Role/Social: Emotional	100 [83 – 100]	100 [66 – 100]	0.296	100 [66 – 100]	100 [100 – 100]	1.00	0 [-16 – 16]	0.969
Role/Social: Physical	66 [33 – 100]	100 [75 – 100]	**0.039***	66 [66 – 100]	100 [66 – 100]	0.221	18 [0 – 37]	0.051
Bodily Pain	80 [40 – 90]	80 [60 – 95]	**0.015***	60 [60 – 80]	80 [20 – 100]	0.766	-1 [-37 – 34]	0.942
Behaviour	83 [58 – 92]	79 [67 – 92]	0.899	75 [50 – 100]	83 [67 – 100]	**0.040***	-7 [-15 – 2]	0.109
Global Behaviour item	85 [60 – 100]	85 [60 – 100]	0.228	85 [60 – 85]	85 [60 – 85]	0.180	-2 [-10 – 6]	0.645
Mental Health	83 [58 – 92]	83 [67 - 92]	0.465	83 [75 – 92]	83 [75 – 92]	0.235	-10 [-22 – 2]	0.114
Self Esteem	75 [60 – 83]	71 [58 – 82]	0.176	83 [75 – 92]	75 [67 – 83]	0.196	9 [2 – 15]	0.914
General Health perceptions	25 [0 – 41]	29 [19 – 50]	0.067	29 [17 – 50]	33 [25 - 42]	0.590	1 [-17 – 19]	0.914
Change in Health	4 [3 - 5]	5 [4 – 5]	**0.049***	3 [2 – 5]	4 [2 – 5]	0.723	0 [-1 – 2]	0.953
Parental Impact: Emotional	63 [50 – 88]	75 [53 – 97]	**0.022***	75 [50 - 88]	75 [50 – 88]	0.877	3 [-20 – 25]	0.812
Parental Impact: Time	100 [75 – 100]	100 [100 - 100]	0.172	100 [83 – 100]	100 [100 – 100]	0.276	-12 [-29 – 5]	0.158
Family Activities	100 [75 – 100]	100 [91 – 100]	**0.010***	98 [88 – 100]	100 [75 – 100]	0.831	2 [-12 – 16]	0.743
Family Cohesion	85 [60 – 85]	85 [60 - 96]	**0.026***	85 [60 – 85]	85 [69 – 85]	0.496	13 [1 – 25]	**0.036***
**Fear of exercise**								
Fear Score children	0 [0 – 2]	0 [0 – 0]	0.066	2 [2 – 3]	0 [0 – 3]	0.131	0 [-1 - 1]	0.843
Fear Score Parents	0 [0 – 1]	0 [0 – 0]	0.109	0 [0 – 3]	0 [0 – 2]	0.102	1 [0 – 1]	0.121

CHQ (child health questionnaire), Values are shown in mean ± SD or as median [IQR]. P-values were measured using paired T-testing (parametric data) or Wilcoxon signed ranks test (non-parametric data). *= P<0.05

Supplement table 5: Disease activity

	Exercise Period (n=15)			Control Period (n=7)				
	Before	After	P-values	Before	After	P-values	Effects size vs controls [95% CI]	P-value Exercise vs control
Fecal calprotectin ($\mu g \cdot g^{-1}$)[1]	400 [57 – 1663]	128 [24 – 642]	**0.016***	1800 [114–3245]	1325 [91–3089]	1.000	-463 [-2016 – 1090]	0.559
ESR ($mm\text{-}hr^{-1}$)	20 [7.0-25]	11 [8 - 21]	0.095	24 [8.0 -29.0]	21.0 [7.0 – 27.0]	0.674	-2.3 [-14.8 – 10]	0.719
CRP ($mg \cdot L^{-1}$)	2.1 [0.7 – 8]	1.2 [0.5 - 6]	0.248	1.8 [0.7 – 9.0]	1.7 [0.6 – 10.0]	0.610	-5.8 [-17.1 – 5.6]	0.320
PCDAI (n=10)	15 [2.5 – 30]	2.5 [0 – 5.0]	**0.012***	15 [5.6 -16.9]	22.5 [5.6 – 45]	0.180	-19.7 [-36 - -3.5]	**0.017***
PUCAI (n=5)	12.5 [6.3 – 18.8]	0 [0 – 3.8]	0.063	30 [20 – 35]	15 [5 – 20]	0.180	-4.1 [-21.3 – 12.9]	0.633
Remission (n)	5	12	**<0.001***	2	2	1.000		**<0.001***
Mild disease activity (n)	7	3	-	3	4	-		
Moderate disease activity (n)	1	0	-	1	1	-		
Severe disease (n)	2	0	-	1	0	-		

ESR (erythrocyte sedimentation rate), CRP (C-reactive protein), PCDAI (Pediatric Crohn's Disease Activity Index), PUCAI (Pediatric Ulcerative Colitis Activity Index), n (number). Values are shown in mean ± SD or as median [IQR]. P-values were measured using paired T-testing (parametric data) or Wilcoxon Rank sum test (non-parametric data). *= P<0.05 *1: P-value compared to outcomes before exercise, 2: Calprotectin (n=13).*

Supplement table 6: Core stability and Muscle strength

	Exercise Period (n=15)			Control Period (n=7)				
	Before	After	P-values	Before	After	P-values	Effects size vs controls [95% CI]	P-value Exercise vs control
**Core stability tests**								
Plank (s)	38 [17 – 58]	77 [47 – 106]	**0.001***	34 [16 - 80]	38 [14 – 84]	0.370	49 [22 – 75]	**<0.001***
Side plank left (s)	13 [9 – 43]	61 [49 – 81]	**0.006***	15 [14 – 63]	13 [12 - 60]	0.337	34 [18 – 50]	**<0.001***
Side plank right (s)	21 [10 - 44]	69 [50 -91]	**0.005***	24 [11 – 51]	26 [12 – 44]	0.103	46 [25 – 67]	**<0.001***
Back bridge (s)	100 [30 – 164]	250 [161 – 358]	**0.001***	130 [70 – 220]	144 [85 – 180]	0.362	153 [101 – 205]	**<0.001***
**Muscle Strength**								
Shoulder abduction (N)	158 [115 – 206]	169 [136 – 211]	0.281	130 [77 – 171]	115 [100 -125]	0.499	20 [-18 – 60]	0.302
Elbow flexion (N)	181 [132 – 210]	176 [149 – 220]	0.427	163 [107 -183]	132 [84 - 179]	0.398	27 [-9 – 64]	0.144
Elbow extension (N)	113 [92 – 162]	133 [111 – 160]	0.609	117 [64 - 156]	92 [64 - 113]	0.176	5 [-35 – 45]	0.819
Squeezing strength (N)	102 [59 – 113]	100 [70.5 – 120]	0.256	74 [57 – 103]	64 [55 – 113]	0.866	6 [-10 – 22]	0.462
Hip flexion (N)	221 [191 - 242]	239 [229 – 269]	**0.020***	168 [122 – 203]	191 [120 – 209]	0.176	25 [2 – 47]	**0.035***
Hip abduction (N)	204 [151 – 210]	198 [180 – 220]	0.140	172 [100 – 189]	159 [98 – 205]	0.735	-4 [-37 – 30]	0.833
Knee flexion (N)	196 [200 – 220]	192 [170 – 228]	0.334	148 [84 – 180]	118 [79 – 168]	0.499	19 [-28 – 66]	0.436
Knee extension (N)	211 [200 – 220]	221 [190 – 231]	0.125	147 [113 – 181]	218 [107 – 223]	**0.043***	-29 [-56 - -1]	**0.043***

s (seconds), N (newton). Values are shown in mean ± SD or as median [IQR]. P-values were measured using paired T-testing (parametric data) or Wilcoxon Rank sum test (non-parametric data). *= P<0.05

Supplement table 7 : Physical activity levels

	Exercise Period (n=15)			Control Period (n=7)				
	Before	After	P-values	Before	After	P-values	Effects size vs controls [95% CI]	P-value difference Exercise vs control
Actigraph								
% of time spent in sedentary (%)	81.0 [75.3 – 86.4]	80.4 [76.6 – 84.3]	0.363	76.9 [71.3 – 82.2]	78.1 [71.9 – 85.7]	0.735	-3.1 [-10 – 3.4]	0.350
% of time spent in MVPA (%)	12.1 [8 – 17.7]	12.1 [9.2 – 15.3]	0.551	15.7 [11.0 – 20.3]	14.2 [9.0 – 18.3]	0.866	2 [-3 – 6.9]	0.435

MET (metabolic equivalent), MVPA (moderate to vigorous physical activity), Values are shown in mean ± SD or as median [IQR]. P-values were measured using paired T-testing (parametric data) or Wilcoxon Rank sum test (non-parametric data). *= P<0.05

Supplement table 8: Body composition, intake and REE.

	Exercise Period (n=15)			Control Period (n=7)				
	Before	After	P-values	Before	After	P-values	Effects size vs controls [95% CI]	P-value difference Exercise vs control
Height (cm)	167.7 [154 – 182]	167.7[157.5 -182]	**0.003***	156 [137 – 171]	157.5 [142.5 – 173]	**0.028***	-1.5 [-3.5 – 0.6]	0.104
Weight (kg)	57.7 [41.7 – 85]	62.1 [41.7 – 83]	**0.036***	54.1 [32.6 – 62.6]	53.2 [35.5 – 57.7]	0.310	2.3 [-5.5 – 10.1]	0.540
**Skinfold Measurements**								
Bodyfat (%)	15.3 [10 – 23.5]	14.9 [9.4 – 25]	0.552	15.6 [8.9 -23.5]	11.7 [7.7 - 20]	**0.028***	2.7 [1.5 – 3.8]	**<0.001**
**BODPOD Measurements**								
Bodyfat (%)	23.1 [14.4 – 37]	27 [17 – 35.6]	**0.041***	27.4 [13.3 – 33.9]	18.7 [15.3 – 37]	0.310	3 [-0.2 – 6.2]	0.064
**Consult dietician**								
REE measured (% predicted)	114 [95 – 125]	106 [101 – 127]	0.865	117 [114 – 121]	105 [93 – 127]	0.398	0 [-0.2 – 0.2]	0.962
Difference TEE and caloric intake (calories)	839 [132 – 1037]	483 [285 – 1088]	0.778	837 [583 – 981]	839 [132 – 1037]	0.173	78 [-96 – 253]	0.202

Centimeters (cm), kilograms (kg), REE (rest energy expenditure), TEE (total energy expenditure). Values are shown in mean ± SD or as median [IQR]. P-values were measured using paired T-testing (parametric data) or Wilcoxon Rank sum test (non-parametric data). *= $P<0.05$
